# Florid cemento osseous dysplasia in association with dentigerous cyst

**DOI:** 10.4103/0973-029X.72503

**Published:** 2010

**Authors:** Karpagaselvi Sanjai, Jayalakshmi Kumarswamy, Vinod K Kumar, Archana Patil

**Affiliations:** *Department of Oral Pathology and Microbiology, Vydehi Institute of Dental Sciences and Research Center, #82, EPIP Area, Nallurahalli, Whitefield, Bangalore - 560 066, Karnataka, India*

**Keywords:** Cemento-osseous dysplasia, familial gigantiform cementoma, fibro-osseous dysplasia (FOD), florid cemento-osseous dysplasia

## Abstract

We present a case of florid cemento-osseous dysplasia occurring in a 20-year-old Indian woman. The subject presented with three lesions involving the maxillary right quadrant, maxillary left quadrant and mandibular left quadrant. The mandibular left quadrant also demonstrated a cyst.The diagnosis was made by correlating the clinical presentation with that of the radiological and histopathological findings. This is a rare entity because of an unusual combination of Asian race along with the association of dentigerous cyst.

## INTRODUCTION

The florid cemento-osseous dysplasia (FCOD) is a category of Cemento - osseous dysplasia (COD) with multifocal involvement of the jaws.[1] Agazi and Beloni reported the first case of gigantiform cementoma,[2] which later came to be known as FCOD in the second edition of WHO’s “International histological classification of odontogenic tumors.”[3] Initially, the FCOD was reported under variety of lesions such as multiple cemento-ossifying fibroma, sclerosing osteomyelitis, multiple enostosis and gigantiform cementoma[4] and many also referred it as paget’s disease of mandible and periapical cemental dysplasia.[5] The term “florid” which refers to the widespread and extensive manifestations of the disease, is characteristic of FCOD and was coined by Melrose *et al*.[2,3,6] The FCOD presents as symmetrical lesions affecting the sextant and sometimes, all the quadrants may be involved. It is seen more commonly in blacks (78%) than in whites (5%) and Asians (4%).[2] In a systematic review done by MacDonald and Jankwoski, the number of reported cases in Indians is three.[3] The females are more commonly affected, with a male:female ratio of 1:2.6.[2] The FCOD commonly occurs in the teeth-bearing regions of the jaw, with a predilection for the posterior region of the mandible (78%).[2]

The pathogenesis of the FCOD has been bestowed on periodontal ligament by most authorities. However, few authors suggest remnants of cementum left in the bone after extraction[2,7] or exposure of such cemental masses to the oral environment by alveolar atrophy under a denture[8,9] also as the reason for the origin of FCOD.

## CASE REPORT

**Figure 1:**
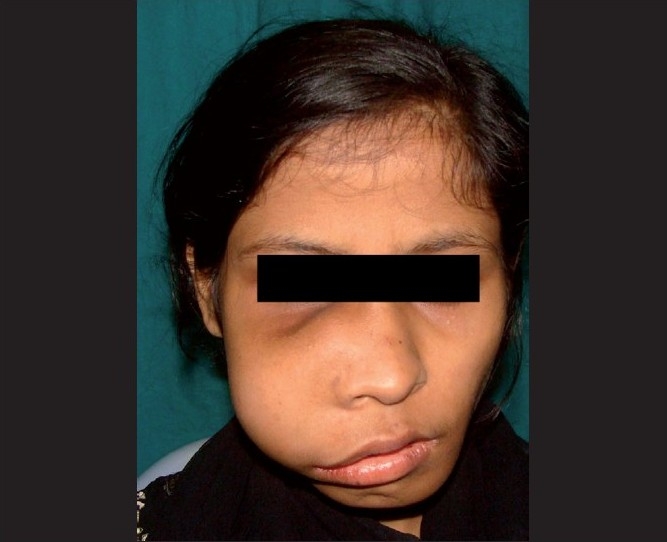
Swelling over the right maxilla and the left mandibular region leading to facial asymmetry

A 20-year-old female patient reported to our institute with a swelling on the zygomatic area on the right side of the face. The swelling was noticed 10 years ago, which remained asymptomatic and had gradually increased to the present size of 6 cm in diameter causing asymmetry of the face [Figure 1]. Another bony hard swelling was also seen in relation to the left mandible extending laterally to the symphysis area [Figure 1]. In both swellings, the color of the surface of the skin was normal and not attached to underlying tissue. There was no local raise in temperature over the swelling.

**Figure 2:**
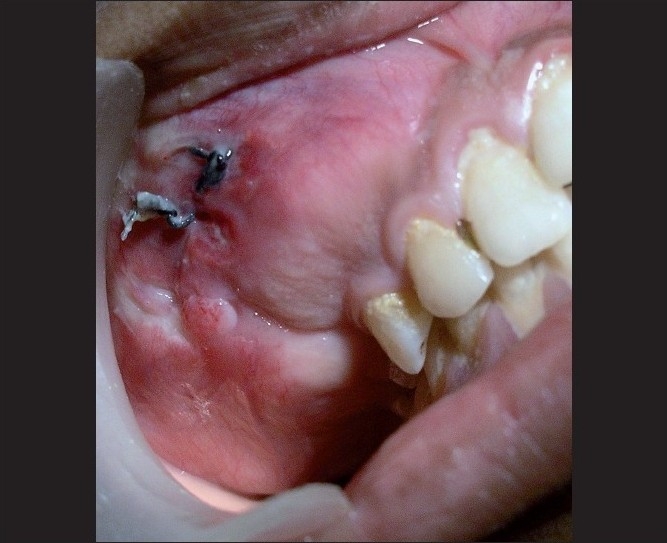
Intraoral involvement of the maxillary right quadrant

**Figure 3:**
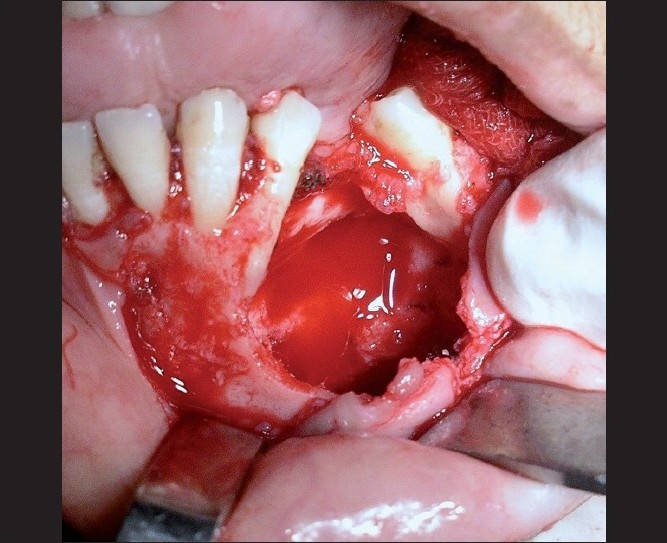
Intraoral picture of the left mandibular quadrant showing surgical exposure of the lesional site extending from 33 to the mesial root of 36

The intraoral examination in relation to the maxillary right quadrant revealed a bony hard swelling involving the buccal [Figure 2] and palatal aspects of the canine area, extending up to the maxillary tuberosity. The maxillary left quadrant revealed a bony hard swelling involving the palatal aspect of the premolar extending up to the molar region. The mandibular left quadrant revealed a hard swelling leading to obliteration of the vestibular sulcus from the canine to the first molar region [Figure 3].

**Figure 4:**
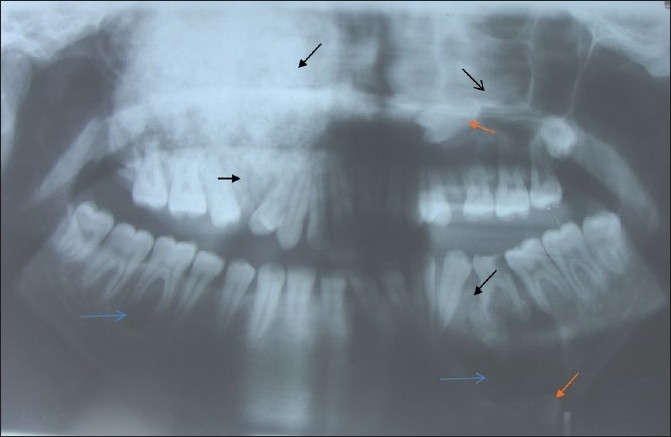
Orthopantomograph: black arrows showing radiopacities, blue arrows showing radioluscent areas and orange arrows showing the impacted premolar

The orthopantomograph of the maxillary right quadrant revealed mixed, radiopaque [Figure 4 black arrows] and radiolucent areas [Figure 4 blue arrows] with diffuse, ill-defined margins obliterating the maxillary sinus, extending from the infraorbital region to the alveolar region. The medial aspect revealed opacity extending into the nasal cavity and laterally extending to the zygomatic arch and posteriorly up to the maxillary tuberosity.

The maxillary left quadrant revealed an impacted second premolar [Figure 4 orange arrow] present at the apex of the first premolar and radiopacity [Figure 4 black arrows] was seen surrounding the impacted premolar. The mandibular left quadrant showed radiopacity [Figure 4 black arrows] in relation to apical areas of the mesial and distal roots of the retained deciduous second molar. In addition, the mandibular left quadrant demonstrated a unilocular radiolucent area with sclerotic margin in relation to the premolar and molar regions extending to the crown of the impacted second premolar at the lower border of the mandible [Figure 4 orange arrows]. The mandibular right quadrant demonstrated unilocular radiolucency in relation to the molar area [Figure 4 blue arrow]. The complete blood picture and serum alkaline phosphatase was performed and was found to be within the normal range.

On the basis of clinical and radiological findings, dentigerous cyst was suspected involving the impacted 35. Incisional biopsy was performed on the maxillary right quadrant and provisional diagnosis of cemento-ossifying fibroma was given. Surgery was planned and performed for all three quadrants under general anesthesia.

### Macroscopy

**Figure 5:**
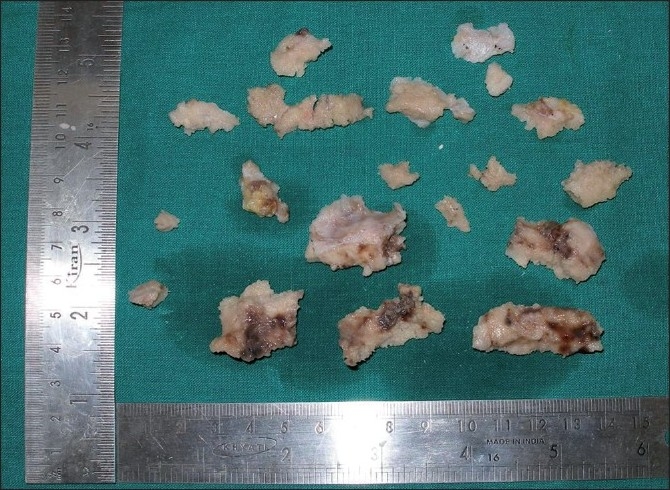
Grossing picture of the maxillary right quadrant

**Figure 6:**
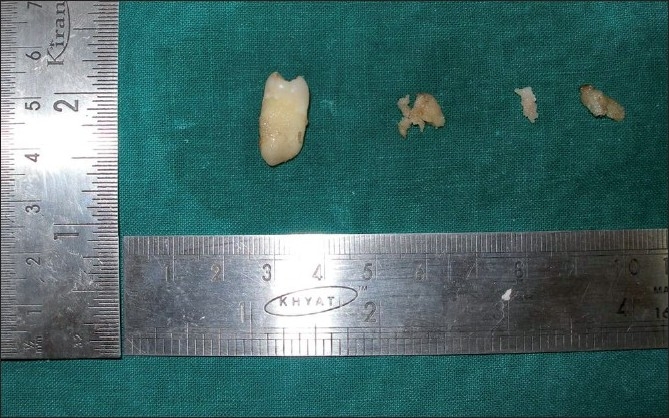
Grossing picture of the maxillary left quadrant showing impacted 25

**Figure 7:**
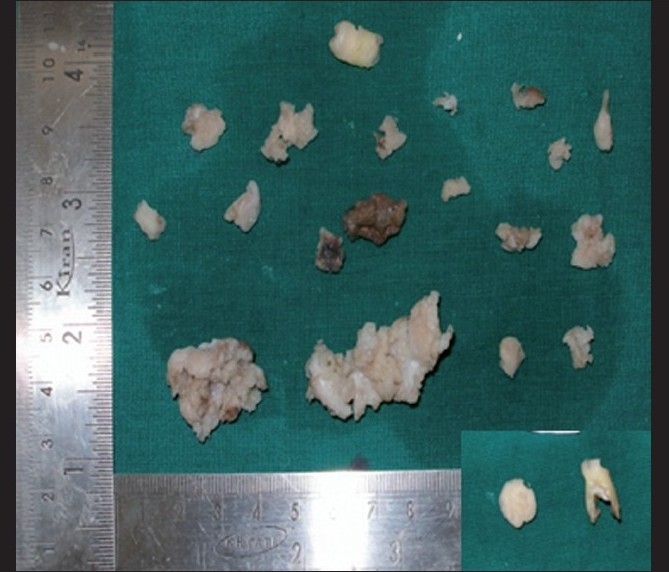
Grossing picture of the mandibular left quadrant and inset shows retained 75 with a well-defined root outline along with a mass

The surgical specimen received showed multiple bits of tissue that were white in color with a gritty surface. It was hard in consistency in relation to the maxillary right quadrant [Figure 5], maxillary left quadrant [Figure 6] and mandibular left quadrant [Figure 7]. In addition to this, an impacted permanent second premolar was seen in relation to the maxillary and mandibular left quadrant. The mandibular left quadrant contained soft tissue, which was firm in consistency, along with a retained deciduous second molar. In case of the retained deciduous molar, the radiographic picture of fused radiopacities at the mesial and distal root apex was not evident as the retained tooth was devoid of such attachments and the roots of the tooth were well defined, as shown in the inset of Figure 7.

**Figure 8:**
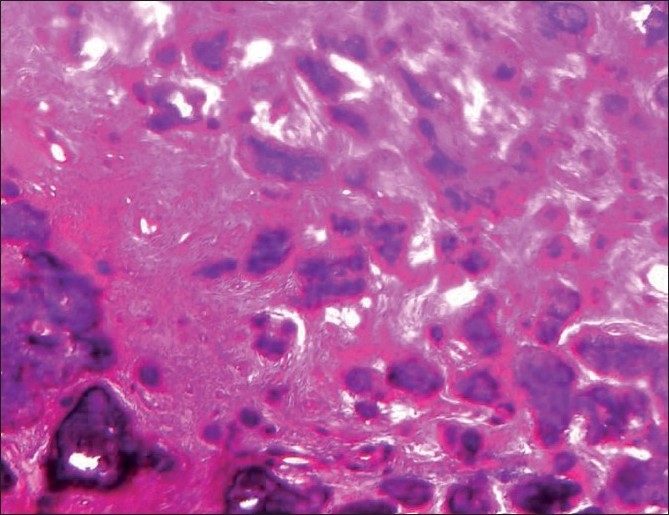
Hematoxylin and Eosin stained section of the maxillary right quadrant showing cemental masses in the cellular connective tissue stroma (4×)

**Figure 9:**
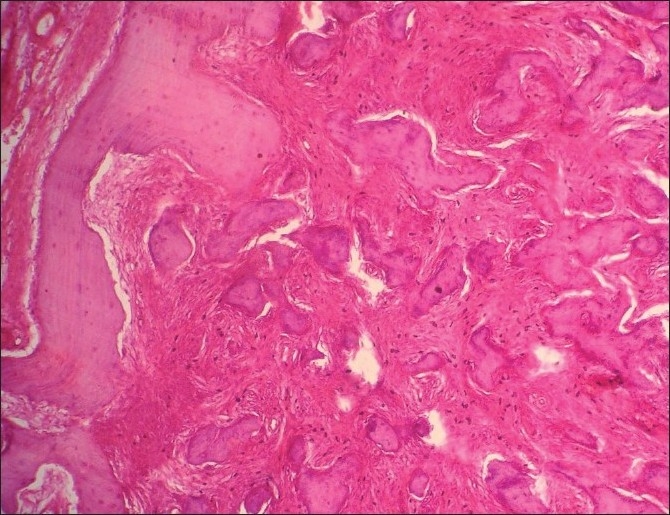
Hematoxylin and eosin stained section of the maxillary right quadrant showing cemental masses fusing with the trabeculae of the bone (10×)

Microscopic features in relation to the maxillary right quadrant exhibited trabeculae of immature bone and irregular basophilic cemental masses in a moderately cellular fibrous connective tissue stroma [Figure 8]. The periphery of the section exhibits normal vital bone. There is no connective tissue separating the immature trabeculae from the normal bone. The cemental masses appear to be fusing with the trabeculae of the bone and with themselves to form larger masses [Figure 9].

**Figure 10:**
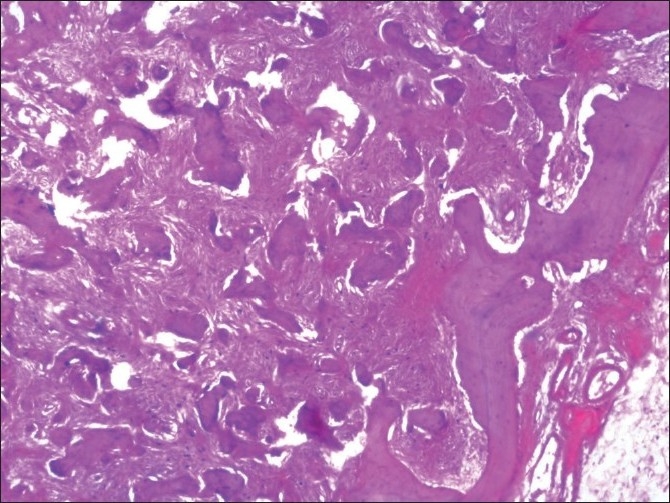
Hematoxylin and eosin-stained section of the maxillary left quadrant showing cemental masses in the connective tissue stroma (4×)

**Figure 11:**
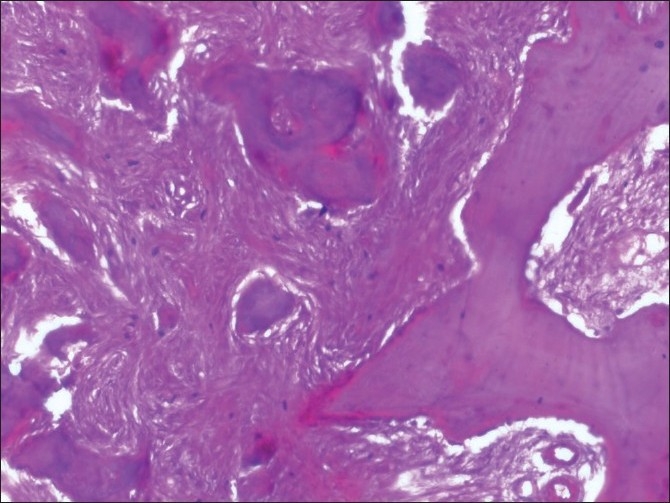
Hematoxylin and eosin-stained section of the maxillary left quadrant showing higher magnification of the previous slide (20×)

The decalcified tissue section in relation to the maxillary left quadrant showed trabeculae of immature bone and irregular basophilic cemental masses in a highly cellular fibrous connective tissue stroma exhibiting whorling around the cemental masses and the immature bone [Figure 10]. The immature bone appears to be curvilinear, resembling ginger roots. Cemental masses appear to be fusing with trabeculae of the bone and with themselves to form larger masses [Figure 11].

**Figure 12:**
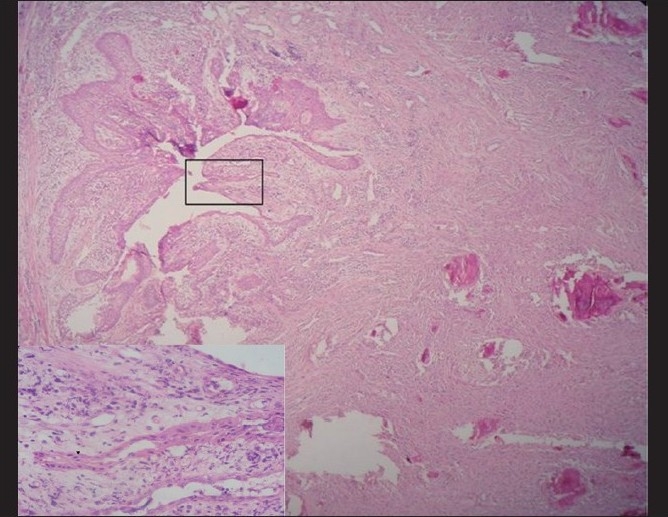
Hematoxylin and eosin-stained section (4×) showing dentigerous cyst in the mandibular left quadrant in continuity with the connective tissue stroma having cemental masses. Inset shows higher magnification (40×) of the cyst epithelium resembling the reduced enamel epithelium

**Figure 13:**
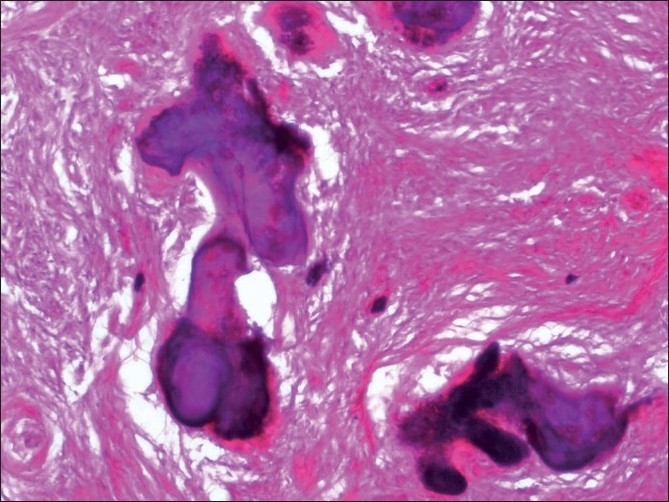
Hematoxylin and eosin-stained section of the mandibular left quadrant showing cemental masses in the connective tissue stroma (10×)

The mandibular left quadrant showed a cyst-like area with irregular basophilic cemental masses in a background of less-cellular fibrous connective tissue [Figure 12]. The cystic lumen is lined by non-keratinized odontogenic epithelium resembling reduced enamel epithelium [inset; Figure 12]. The epithelium shows proliferation, with the underlying connective tissue being infiltrated with lymphocytes and plasma cells. Other areas showed fibrous connective tissue stroma with cemental masses [Figure 13].

## DISCUSSION

The cement-osseous dysplasia is considered as a non-neoplastic fibro-osseous lesion wherein bone is replaced by fibrous connective tissue along with the cemental masses and is categorized into three different entities, such as periapical, focal and florid. However, now the COD term has been replaced as osseous dysplasia in the recent WHO classification and is believed that the three entities represent a spectrum of lesions appearing to be reactive in nature and have differences only in clinical and radiological presentation. Histologically, it appears to affect the tooth-bearing areas probably derived from the periodontal ligament and from the cementum or cemental-like tissues.[7]

The periapical osseous dysplasia appears as a radiolucent area at the apices of the anterior teeth and with time, the mineralization becomes radiopaque[2] and rarely exceeds more than 1 cm,[7] remaining relatively asymptomatic. The focal COD appears to arise at the previous extraction site or at the apices of the molar region, rarely exceeding 2 cm in diameter, with similar radiological presentation as that of the periapical osseous dysplasia. Both these variants may involve a single area or multiple areas in a quadrant. If there is symmetrical presentation affecting more than one quadrant, then the diagnosis of FCOD is considered.[7] If there is a familial basis to the florid-osseous dysplasia, the familial gigantiform cementoma can be considered.[2,7]

Although histopathology will remain similar for all the types, as this represents a spectrum of lesion rather than an entity,[7] the early lesion represents cellular fibrous tissue containing trabeculae of woven bone with cementum-like calcification.[7] With maturation, the ratio of fibrous tissue to mineralized materials are decreased and trabeculae become more curvilinear structures[1] that progress to the radiopaque stage in which the cementum-like tissue coalesce to form large basophilic calcification with resting and reversal lines.[1] There could be some difference in stage or degree of calcification, which will be in hand with radiological presentation. In the present case, the maxillary right quadrant resembled the most mature lesion, with large basophilic cemental masses [Figure 8] resembling a radiopaque stage, whereas the maxillary left and mandibular left quadrant resembled early to mature stages of lesion [Figures 10, 11 and 13], with a difference in the degree of calcification, which was evident as radiopacities in the radiograph [Figure 4].

The diagnosis of COD is a challenge, especially when it shows the cortical plate enlargement along with mixed radiopacities, as seen in the present case, in relation to the maxillary right quadrant [Figures 2 and 4] and may resemble Pagets disease of bone. Thus, Paget’s disease of bone may remain as differential diagnosis for COD[1,10–12] and has to be ruled out as the former may transform into osteosarcoma.[1] The dysplastic lesion of Paget’s is always polyostotic[10] and because this case did not show an increase in the serum alkaline phosphatase level and in histopathology, there was no jigsaw puzzle or mosaic pattern of trabeculae seen, ruling out Paget’s disease. The second most common lesion that interfered with the diagnosis of COD was the fibrous dysplasia.[12] However, fibrous dysplasia exhibits a ground glass pattern rather than a cotton wool appearance and histopathology of the specimen tissue exhibited irregular basophilic globular cemental masses that are not seen in fibrous dysplasia. Hence, the possibility of fibrous dysplasia could be ruled out.

The present case showed connective tissue fibers as filling material between the trabeculae, which may resemble chronic sclerosing osteomyelitis.[10,12] Sequestra and periosteal bone formation were absent in the radiological picture. There was no clinical history of pain or pus drainage nor evident sinus tract in relation to the lesional site. The histopathology of the lesional tissue does not show areas of abscess formation, ruling out the possibility of chronic sclerosing osteomyelitis.

The histopathology of COD most commonly resembles cemento-ossifying fibroma.[1,2,7] Cemento-ossifying fibroma, although not always, shows characteristic connective tissue capsule surrounding the lesional tissue, but is well demarcated grossly and microscopically and therefore is most often received as intact specimen.[1,7] But, the COD is received as multiple bits as it is not demarcated from the surrounding bone. Because the biopsy specimen of the present case was received in multiple bits, it is in accordance with gross picture of COD. The trabeculae and cemental masses in cemento-ossifying fibroma most commonly blend with that of connective tissue whereas in case of COD, the trabeculae or the cemental masses will be retracted from the surrounding connective tissue,[1] which is also seen in this case. Therefore, the diagnosis is less likely to be cemento-ossifying fibroma.

Because this case demonstrated impaction of permanent premolars in the maxillary left and mandibular left quadrants, the possibility of Gardner’s syndrome has to be questioned as earlier reports suggested misdiagnosing Gardner’s syndrome as COD.[10] However, as skeletal, skin manifestations and intestinal polyps of Gardner’s syndrome were not seen, it eliminates the possibility of Gardner’s syndrome.

The mandibular left quadrant showed radiopacity in relation to the apex of the root surface of the retained deciduous molar. However, the received specimen did not show any attachment to the apex of the root, a feature of cementoblastoma.[6] The present case demonstrated the cemento-osseous tissue with the absence of fusion to the apex of the tooth root, which rules out the possibility of cementoblastoma.

In addition, the mandibular left quadrant also showed unilocular radiolucency that resembled a dentigerous cyst in histopathological findings. However, earlier reports suggested FCOD in association with simple bone cyst,[2] which exhibits a cystic cavity not lined by a cystic epithelium. As the present case consisted of cystic lining epithelium, the possibilities of it being simple bone cyst are ruled out. To our review, this is the first case of FCOD with cystic lining epithelium to be reported.

## CONCLUSION

We conclude this case as an unusual manifestation of FCOD in association with dentigerous cyst in a 20-year-old woman of Asian race. There was no relationship between dentigerous cyst and FCOD. This could be considered as the first case of dentigerous cyst co-existing with FCOD. Most often, the cases of FCOD should not be surgically treated, as it may result in secondary osteomyelitis. But in the present case, surgery was performed as it caused asymmetry of the face. In such a situation, close monitoring of the patient is necessary to prevent secondary complications of osteomyelitis.
